# Manifestations and Virus Detection in the Ocular Surface of Adult COVID-19 Patients: A Meta-Analysis

**DOI:** 10.1155/2021/9997631

**Published:** 2021-06-19

**Authors:** Yu-Yen Chen, Yung-Feng Yen, Li-Ying Huang, Pesus Chou

**Affiliations:** ^1^Department of Ophthalmology, Taichung Veterans General Hospital, Taichung 407, Taiwan; ^2^School of Medicine, National Yang-Ming University, Taipei 112, Taiwan; ^3^Community Medicine Research Center and Institute of Public Health, National Yang-Ming University, Taipei 112, Taiwan; ^4^School of Medicine, Chung Shan Medical University, Taichung 402, Taiwan; ^5^Section of Infectious Diseases, Taipei City Hospital, Taipei 103, Taiwan; ^6^Institute of Hospital and Health Care Administration, National Yang-Ming University, Taipei 112, Taiwan; ^7^Department of Health Care Management, National Taipei University of Nursing and Health Sciences, Taipei 112, Taiwan; ^8^School of Medicine, College of Medicine, Fu Jen Catholic University, New Taipei 242, Taiwan; ^9^Division of Endocrinology and Metabolism, Department of Internal Medicine, Fu Jen Catholic University Hospital, New Taipei 243, Taiwan

## Abstract

**Purpose:**

This study aims to examine the prevalence rate of ocular manifestations and the positive rate for the real-time reverse transcriptase-polymerase chain reaction (RT-PCR) of severe acute respiratory syndrome coronavirus 2 (SARS-CoV-2) in conjunctival/tear swabs among adult patients with coronavirus disease 2019 (COVID-19).

**Methods:**

PubMed and EMBASE were reviewed between December 1, 2019, and January 31, 2021, and only peer-reviewed clinical studies in our pooled analyses were included. Details regarding the patient numbers, demographics, ocular manifestations, positivity of ocular surface RT-PCR, and severity of pneumonia were recorded from each study. Primary outcomes were the occurrence of ocular manifestations and virus detection on the ocular surface. Meanwhile, secondary outcomes included frequencies of various ocular symptoms/signs (s/s), the proportion of patients with ocular manifestation as the initial symptom, and the relationship between the severity of pneumonia and the presentation of ocular manifestations.

**Results:**

In total, 35 studies with 4,432 adult COVID-19 patients were included in this analysis. The overall prevalence rate of ocular manifestations was found to be 11.3%, and the positive rate of SARS-CoV-2 in the ocular surface was 7.4%. The four most common ocular s/s were follicular conjunctivitis, redness, watering, and discharge. A proportion of 3.3% presented with ocular s/s preceding other findings. Besides, patients with higher severity of pneumonia were more likely to have ocular manifestations (odds ratio = 2.25; 95% confidence interval (CI): 1.45–3.50).

**Conclusion:**

As per our findings, it was determined that ocular transmission of SARS-CoV-2 might be possible, highlighting the importance of eye protective equipment among healthcare personnel.

## 1. Introduction

Coronavirus disease 2019 (COVID-19) is caused by the novel severe acute respiratory syndrome coronavirus 2 (SARS-CoV-2). It was recognized by the World Health Organization (WHO) as a pandemic in March 2020. By the end of February 2021, more than 100 million cases have been confirmed, and more than 2.2 million deaths have been recorded globally.

SARS-CoV-2 has been determined to bind to the angiotensin-converting enzyme 2 (ACE2) receptor, which is mostly located in the lung, heart, gastrointestinal tract, and kidney [1, 2]. Therefore, the most common symptoms/signs (s/s) are dyspnea, fever, diarrhea, and heart/renal failure. The conjunctiva has a lower concentration of ACE2 receptors, is exposed to the environment, and is easily contacted with the respiratory droplets or hands capable of carrying viruses. Therefore, the conjunctiva may be an infection route of SARS-CoV-2 [3, 4]. Some COVID-19 patients present with conjunctivitis as symptoms of redness, watering, discharge, foreign body sensation, chemosis, etc. However, the prevalence rate of ocular manifestations was found to differ a lot among different studies [5, 6].

Except for the findings of ocular s/s, the direct evidence of ocular involvement by SARS-CoV-2 is the detection of the virus RNA in the ocular surface. Real-time reverse transcriptase-polymerase chain reaction (RT-PCR) assays are currently the standard for COVID-19 diagnosis. Data published on the topic of ocular surface involvement presented as the results of RT-PCR in conjunctival swabs or tear samples remain to be inconsistent [5]. These studies have been conducted in a hospital setting, mostly with small case numbers. To better identify the ocular surface manifestations and analyze the positive rate for the SARS-CoV-2 RT-PCR in the ocular surface among adult COVID-19 patients, a meta-analysis of relevant studies was conducted. Whether COVID-19 patients with more severe pneumonia would have a higher prevalence of ocular manifestations was also examined.

## 2. Materials and Methods

### 2.1. Search Strategy

This study was conducted in accordance with the Preferred Items for Systematic Reviews and Meta-Analyses (PRISMA) guidelines. PubMed and EMBASE were reviewed for studies published from December 2019 to February 2021, using the keywords “(SARS-CoV-2 or COVID-19 or 2019-nCoV) and (conjunctiva or conjunctivitis or ocular manifestations or conjunctival swab or ocular symptoms).” The titles and abstracts of the studies were first screened. Candidate papers were further scrutinized in their full texts to see if they have fulfilled the inclusion criteria. Bibliographies were also manually searched for relevant literature.

### 2.2. Inclusion and Exclusion Criteria

Only peer-reviewed journal articles written in English were included in this analysis. The confirmation of COVID-19 cases should be based on the clinical criteria or positive RT-PCR for virus RNA from nasopharyngeal swabs. These studies should be cross-sectional, prospective, or retrospective clinical cohort studies or case series. Studies or case reports with case numbers not larger than five were excluded. Reviews, meta-analyses, conference abstracts, or letters to editors were also excluded due to possible repeated cases. Two researchers (Chen and Yen) independently assessed the eligibility of these articles. A third researcher (Huang) reassessed and determined the eligibility if discrepancies occurred.

Evaluation of the quality of included studies was independently performed by three researchers (Chen, Yen, and Huang) using Hoy's checklist [7]. Consensus should be reached. If not, the supervisor (Chou) would make the final decision. The checklist assesses the risk of bias on 9 domains, including representativeness of the target population, random selection, likelihood of nonresponse bias, data collection, use of case definitions, reliability and validity of measuring tools, and appropriate use of numerator and denominator for the ocular symptoms. Each domain contains a question (criteria). The domain was rated as “Yes” and scored 0 if the criteria were satisfied. On the contrary, the domain was rated as “No” and scored 1 if the criteria were not fulfilled. Then, the total score was obtained by summation of the 9 numbers to decide the risk of bias. The risk of bias was classified as low if the total score was 0–3, moderate if the total score was 4–6, and high if the total score was more than 6.

### 2.3. Extraction of Variables

The following data were recorded from each included article: the first author, date of publication, mean age of participants, total number of patients, clinical features of ocular s/s, and the number of patients who had ocular s/s, who had positive conjunctival RT-PCR, and who had ocular s/s as the first presentation of the disease. Additionally, proportions of patients who had ocular s/s among those with severe pneumonia and mild-to-moderate pneumonia were also collected.

### 2.4. Statistical Analysis

The Comprehensive Meta-Analysis software, version 3 (Biostat, Englewood, NJ, USA), was used to perform the meta-analysis. The primary outcomes are as follows: (1) the proportion of patients with ocular s/s among COVID-19 cases and (2) the positive rate of RT-PCR from conjunctival/tear samples among COVID-19 cases. The secondary outcomes included the following: (1) clinical features of ocular manifestations, (2) subgroup analysis regarding proportions of the four most common ocular s/s, (3) the proportion of patients whose first s/s appeared as ocular manifestations, and (4) the odds ratio (OR) for ocular s/s among patients with severe pneumonia relative to mild-to-moderate pneumonia.

Besides, between-trial heterogeneity of the primary outcomes was calculated using *I*^2^ statistics. *I*^2^ statistics of ≥75% represents considerable heterogeneity. Publication bias was determined using funnel plots and Egger's test.

## 3. Results

### 3.1. Search Results

**Figure 1:**
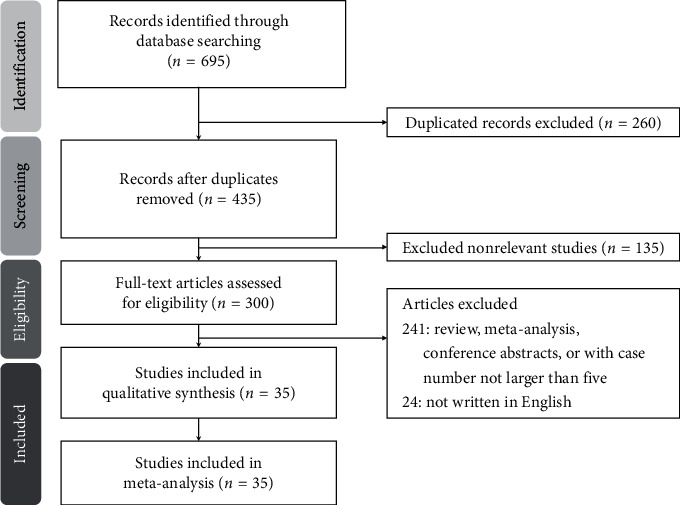
Preferred reporting items for systematic reviews and meta-analyses (PRISMA) flow diagram for the searching and identification of included studies.

Figure 1 illustrates the PRISMA flowchart of study screening and selection. An initial search yielded 695 citations. Of them, 260 were removed because of duplication. Subsequently, 135 nonrelevant studies were excluded after screening titles and abstracts. Following full-text examination, 241 studies categorized as reviews, meta-analyses, or case reports with case numbers not larger than five were also excluded. Furthermore, another 24 papers not written in English were excluded. Finally, 35 studies with a total of 4,432 patients were included in our meta-analysis.

### 3.2. Evaluation of the Quality of Included Studies

The risk of bias of each study is presented in Table 1. Most of them had a moderate risk of bias and none of them had a high risk of bias.

### 3.3. Characteristics of Included Studies

Table 2 summarizes the characteristics of 35 studies. Ten of them were conducted in China. Most of the patients were older than 40 years. Due to the infection risk of COVID-19, a thorough examination with a slit lamp or ophthalmoscopy was rarely performed. Most of the studies retrieved the s/s by questionnaires, by simplified ocular examination (torch, portable slit lamp, etc.), or from chief complaints of the patients. The patients showed no ocular s/s in five studies. Of the 35 studies, 21 evaluated the positivity of RT-PCR for virus RNA in ocular conjunctival/tear samples, and most of them revealed a positive rate of less than 10%.

### 3.4. Primary Outcomes

**Figure 2:**
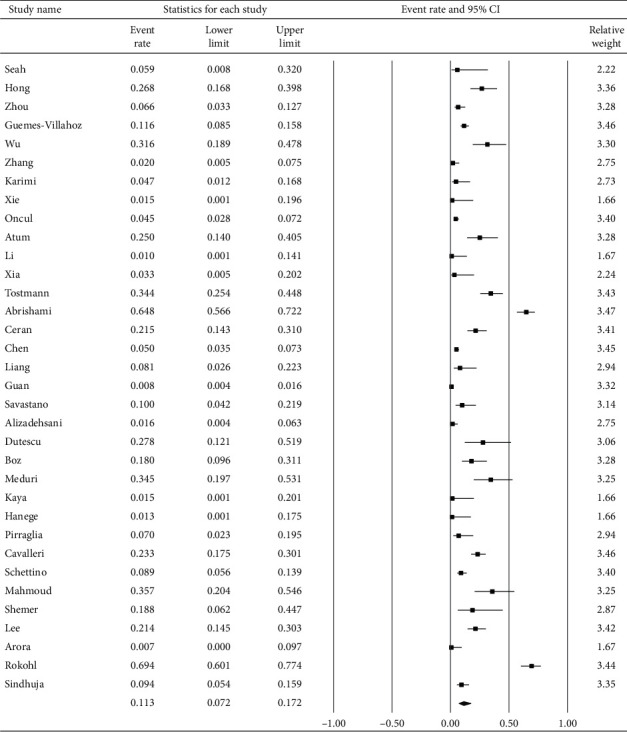
The overall prevalence of ocular manifestations of included studies.

**Figure 3:**
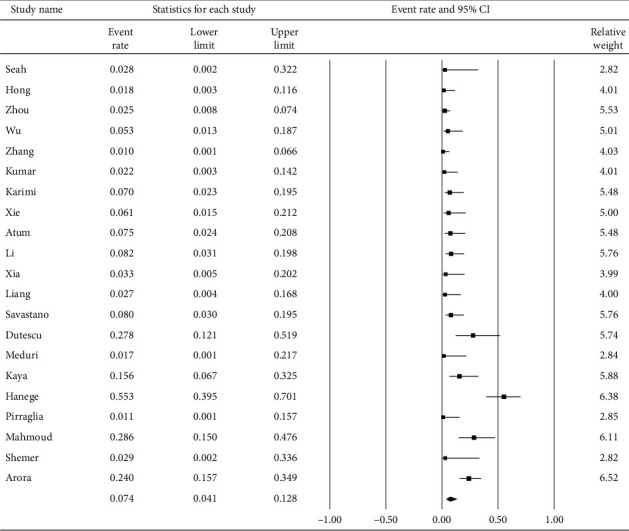
The overall positive rate of RT-PCR for SARS-CoV-2 in conjunctival/tear samples.

Figure 2 demonstrates that 11.3% (95% confidence interval (CI): 7.2–17.2%) of COVID-19 patients had ocular s/s using random-effects model from pooling of the 30 studies.  Figure 3 illustrates that using the same model, the overall positive rate of RT-PCR for the virus in the ocular surface from the 21 studies was 7.4% (95% CI: 4.1–12.8%).

### 3.5. Heterogeneity and Publication Bias

**Figure 4:**
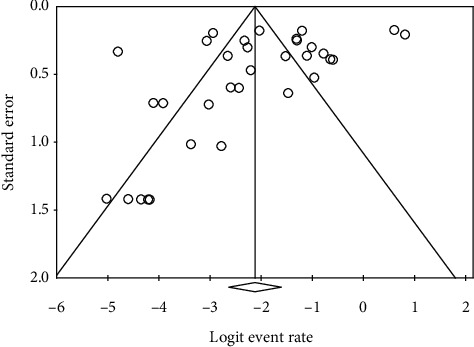
Funnel plot of studies regarding the proportion of ocular manifestations.

**Figure 5:**
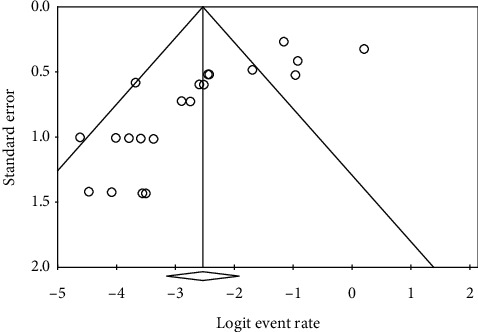
Funnel plot of studies regarding the positive rate of ocular surface RT-PCR for SARS-CoV-2.

Considerable heterogeneity in studies was found in evaluating the proportions of patients having ocular s/s and the positive rate of ocular surface RT-PCR (*I*^2^ = 94.4% and 80.3%, respectively). The funnel plots for calculating publication bias are presented in Figures 4 and 5. The results of Egger's test revealed *p*=0.12 and *p* < 0.0001, respectively.

### 3.6. Sensitivity Analysis

The study conducted by Tostmann et al. had different inclusion criteria from other studies because it included healthcare workers only. When the study was removed from the analysis, the remaining studies yielded an overall proportion of ocular manifestations to be 10.7% (95% CI: 6.7–16.7%). Considerable heterogeneity still existed (*I*^2^ = 94.5%).

### 3.7. Secondary Outcomes

Table 3 shows the clinical features of the ocular manifestations. Classification of ocular manifestations included redness, follicular conjunctivitis, watering, discharge, dryness, and itching. Proportions of patients whose first s/s were ocular manifestations varied a lot from 0% to 31.3%.

**Figure 6:**
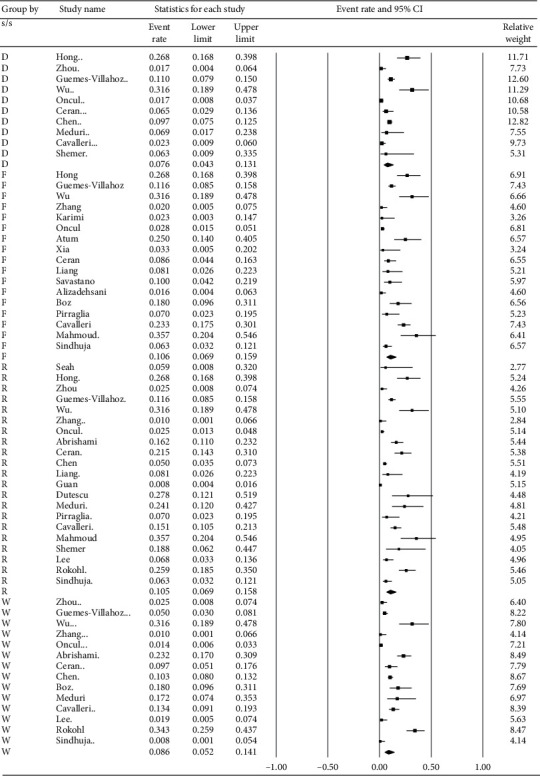
Subgroup analysis regarding the four most prevalent ocular symptoms/signs. D represents discharge. F represents follicular conjunctivitis, R represents redness, and W represents watering.

**Figure 7:**
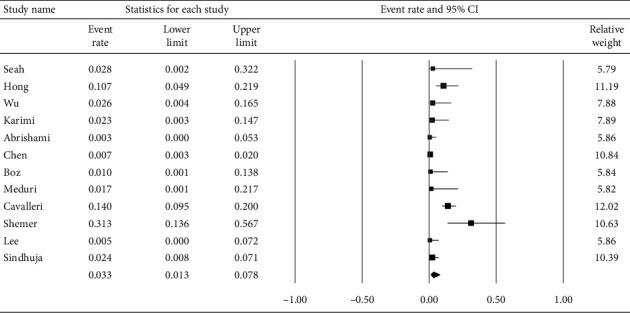
Proportion of patients who had ocular symptoms/signs as the first manifestation of COVID-19.

**Figure 8:**
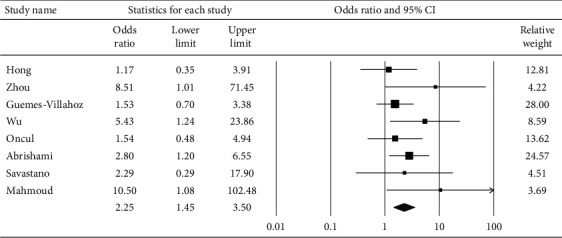
Pooled odds ratio for ocular manifestations among severe pneumonia relative to mild-to-moderate pneumonia.

Figure 6 displays the subgroup analysis according to the four most prevalent ocular s/s, which were follicular conjunctivitis, redness, watering, and discharge. Using random-effects models, the overall prevalence rates were calculated as 10.6%, 10.5%, 8.6%, and 7.6%, respectively (all *p* < 0.001).  Figure 7 reveals the proportion of patients whose first s/s were ocular manifestations. The overall proportion was 3.3% (1.3–7.8%).  Figure 8 explores the relationship between odds of ocular manifestations and severity of COVID-19. Patients with severe pneumonia had significantly higher pooled odds for ocular s/s than those with mild-to-moderate pneumonia (OR = 2.25; 95% CI: 1.45–3.50).

## 4. Discussion

This meta-analysis focused on the ocular manifestations and positive rate of conjunctiva/tear RT-PCR in adult patients with COVID-19. In total, 4,432 patients in 35 studies were included in our analyses. The overall prevalence rate of ocular manifestations was 11.3% (95% CI: 7.2–17.2%), with considerable heterogeneity between studies. Moreover, the overall positive rate of ocular surface RT-PCR was 7.4% (95% CI: 4.1–12.8%) with considerable heterogeneity. The four most prevalent ocular manifestations were follicular conjunctivitis (10.6%), redness (10.5%), watering (8.6%), and discharge (7.6%). A proportion of 3.3% of COVID-19 patients presented with ocular manifestations as their first s/s. Moreover, patients with higher severity of pneumonia had a significantly higher possibility of ocular manifestations.

Our study found an overall proportion of ocular manifestations among COVID-19 patients to be 11.3%, which was compatible with previous meta-analyses performed by Aggarwal et al. [5] and Inomata et al. [6]. Conjunctivitis, presented as redness, watering, discharge, foreign body sensation, etc., can occur in COVID-19 but may go unnoticed due to several reasons. First, doctors may pay more attention to the damage of vital organs (e.g., lung, heart, and kidney) and ignore the ocular findings. Second, detailed ophthalmic examinations, with a short working distance, are not safe to be performed in the acute stage of COVID-19. Third, patients under ventilators frequently have ocular complications, including dryness, chemosis, and subconjunctival hemorrhage. Whether these manifestations are directly caused by SARS-CoV-2 is hard to tell. Fourth, some ocular symptoms are related to previous long-term disorders (e.g., dry eye syndrome, chronic inflammation, or allergy). To address these difficulties regarding data retrieval, most studies used questionnaires and focused on the new-onset s/s during the infection of SARS-CoV-2. Information bias might occur because of differences in subjective perception. Therefore, some studies required a simplified ocular examination (torch, ophthalmoscopy, and photography) in a long distance. However, the carefulness of inspection might influence the sensitivity and specificity. It may explain the considerable heterogeneity between studies, reaching 94.4% in our analysis, which is a limitation of our study. Further studies with a standardized method to record ocular s/s are warranted.

A more straightforward method to indicate virus infection in the ocular surface is RT-PCR analysis of conjunctival secretions/tears. The conjunctiva is easily exposed to the pathogens in the environment and is also connected to the respiratory tract through the nasolacrimal duct. Whether the virus travels from the conjunctiva to the lung or spreads retrogradely from the lung to the conjunctiva remains a concern under discussion. Another issue worth exploring is the inconsistent results of the ocular surface RT-PCR among different studies. Four studies conducted by Seah, Meduri, Pirraglia, and Shemer revealed zero positive rates [8, 31, 34, 38]. However, 21 (55.3%) out of 38 COVID-19 patients in Hanege's study had positive RT-PCT results in conjunctival swabs [33]. The considerable heterogeneity, as high as 80.3%, may be due to variations in techniques and time of collecting samples, the severity of diseases, and testing regimen among different studies. The positivity of RT-PCR in conjunctiva or tears was found to be high when sampled from the 4th to 9th day of symptoms in Arora et al.'s study [40] and then decreased from the second week and beyond in Seah et al. and Zhang et al.'s studies [8, 13]. However, Hu et al. and Colavita et al. found that the positivity remained for over 2 weeks even after nose/pharyngeal swabs had become negative [43, 44]. Therefore, performing RT-PCR in conjunctival/tear swabs at multiple stages (early and late) of infection would be better to reduce the false-negative rate. Besides, the conjunctiva may have a lower viral concentration and a different genome composition compared to the nasopharyngeal specimen [13, 45], highlighting the importance of accurate sampling and appropriate PCR primers. Furthermore, the drainage of the tear film may decrease the virus load on the ocular surface. Immune activation with an increase in lactoferrin and secretory IgA levels in tears may also contribute to the low RT-PCR positive rate of the conjunctiva/tear samples in our study [46].

The most prevalent s/s of the ocular manifestations in our study are follicular conjunctivitis, redness, watering, and discharge. This finding is similar to the meta-analysis conducted by Aggarwal et al. [5], which revealed that the most common ocular manifestations are pain, discharge, and watering. Our study also revealed that 3.3% of the COVID-19 patients had ocular s/s as the prodromal manifestations. Although the proportion is low, doctors and patients should be aware of the occurrence of ocular manifestations because it might be the sentinel presentation of COVID-19.

The strength of our study was the completeness. Compared to previous meta-analyses, we included more studies and patients. The included studies were conducted from Asia, Middle East, Europe, and Africa. Therefore, our analyses would provide a more comprehensive exploration of the topic of COVID-19. The novelty of our study included our further investigation of the relationship between ocular s/s and the severity of COVID-19. Wu et al. found that the patients with ocular s/s were more likely to have higher levels of neutrophil, procalcitonin, C-reactive protein, and lactate dehydrogenase than those patients without ocular s/s [12]. They also found that patients with ocular manifestations had a higher severity of pneumonia. Our study further calculated the pooled odds for ocular manifestations to be significantly higher in patients with severe pneumonia than those with mild-to-moderate pneumonia (OR = 2.25).

The limitation of our analyses was the heterogeneity among the included studies. There is no standardized procedure regarding ocular examination or ocular surface testing for patients with COVID-19. Therefore, interstudy or intrastudy variation may occur. Moreover, some studies acquired data of symptoms using questionnaires or telephone interviews, which might increase the information bias. Globally accepted protocols of data collection and ocular PCR should be applied in upcoming studies.

## 5. Conclusions

In conclusion, this meta-analysis has demonstrated the possibility of ocular manifestations and virus detection in the ocular surface of COVID-19 patients. It also reminds us that the ocular manifestations might occur as a prodromal finding of COVID-19. Furthermore, our study highlights that severity of pneumonia may be significantly associated with ocular manifestations. Further experimental and clinical research should be conducted to derive more data regarding the COVID-19 infection through the ocular surface route. At present, our study provides evidence implying ocular transmission. Thus, doctors should be aware of the ocular manifestations in patients possibly infected with COVID-19. In addition, wearing protective equipment, including eye goggles, by ophthalmologists is imperative to minimize the risk of infection.

## Figures and Tables

**Table 1:** Risk of bias for individual studies included in the meta-analysis.

Study	D1	D2	D3	D4	D5	D6	D7	D8	D9	Sum	Overall risk of bias
Seah et al. [8]	1	1	1	0	0	0	0	0	1	4	Moderate
Hong et al. [9]	1	1	1	0	0	0	1	0	1	5	Moderate
Zhou et al. [10]	1	0	10	0	0	0	1	0	1	4	Moderate
Guemes-Villahoz et al. [11]	1	1	1	0	0	0	0	0	1	4	Moderate
Wu et al. [12]	1	0	1	0	0	0	1	0	1	4	Moderate
Zhang et al. [13]	1	0	1	0	0	1	0	0	1	4	Moderate
Kumar et al. [14]	1	1	1	0	0	0	0	0	1	4	Moderate
Karimi et al. [15]	1	0	1	0	0	0	1	0	1	4	Moderate
Xie et al. [16]	1	1	1	0	0	0	0	0	1	4	Moderate
Oncul et al. [17]	1	0	1	0	0	0	0	0	1	3	Low
Atum et al. [18]	1	1	1	0	0	0	1	0	1	5	Moderate
Li et al. [19]	1	1	0	0	0	0	1	0	1	4	Moderate
Xia et al. [20]	1	1	1	0	0	0	1	0	1	5	Moderate
Tostamann et al. [21]	1	0	1	1	0	1	0	0	1	5	Moderate
Abrishami et al. [22]	1	0	1	0	0	0	0	0	1	3	Low
Bostanci Ceran et al. [23]	1	0	0	0	0	0	1	0	1	3	Low
Chen et al. [24]	1	0	0	0	0	0	1	1	1	4	Moderate
Liang and Wu [25]	1	1	1	0	0	0	0	0	1	4	Moderate
Guan et al. [26]	0	0	0	1	0	0	1	1	1	4	Moderate
Savastano et al. [27]	1	1	1	0	0	0	0	0	1	4	Moderate
Alizadehsani et al. [28]	1	0	0	0	0	0	1	1	1	4	Moderate
Dutescu et al. [29]	1	1	1	0	0	0	0	0	1	4	Moderate
Boz et al. [30]	1	1	1	0	0	0	0	0	1	4	Moderate
Meduri et al. [31]	1	1	1	0	0	0	1	0	1	5	Moderate
Kaya et al. [32]	1	1	1	0	0	0	1	0	1	5	Moderate
Hanege et al. [33]	1	1	1	0	0	0	1	0	1	5	Moderate
Pirraglia et al. [34]	1	1	1	0	0	0	0	0	1	4	Moderate
Cavalleri et al. [35]	1	0	1	0	0	0	1	0	1	4	Moderate
Schettino et al. [36]	1	0	0	0	0	0	1	1	1	4	Moderate
Mahmoud et al. [37]	1	1	1	0	0	0	0	1	1	5	Moderate
Shemer et al. [38]	1	1	1	0	0	0	1	0	1	5	Moderate
Lee et al. [39]	1	0	1	0	0	0	1	1	1	5	Moderate
Arora et al. [40]	1	1	1	0	0	0	0	0	1	4	Moderate
Rokohl et al. [41]	1	0	1	0	0	0	0	1	1	4	Moderate
Sindhuja et al. [42]	1	0	1	0	0	0	1	0	1	4	Moderate

*D* = dimension; D2: Was the sampling frame a true or close representation of the target population? D3: Was some form of random selection used to select the sample, OR, was a census undertaken? D4: Was the likelihood of nonresponse bias minimal? D5: Were data collected directly from the subjects (as opposed to a proxy)? D6: Was an acceptable case definition used in the study? D7: Was the study instrument that measured the parameter of interest shown to have reliability and validity? D8: Was the same mode of data collection used for all subjects? D9: Were the numerator(s) and denominator(s) for the parameter of interest appropriate?

**Table 2:** Characteristics of patients in studies included in the meta-analysis.

First author	Date in 2020	Type	Country	Study population	Number \of pts	Mean age	Collected s/s	Pts with ocular s/s	Positive ocular RT-PCR
Seah et al. [8]	Mar 24	P	Singapore	Hospitalized	17	37.0^†^	OE	1 (5.9)	0 (0)
Hong et al. [9]	Apr 26	CS	China	Hospitalized	56	48.0	Q	15 (26.8)	1 (1.8)
Zhou et al. [10]	Apr 21	CS	China	Hospitalized	121	48.0^†^	OE	8 (6.6)	3 (2.5)
Guemes-Villahoz et al. [11]	Aug 29	CS	Spain	Hospitalized	301	72.0^†^	OE	35 (11.6)	NA
Wu et al. [12]	Mar 31	CS	China	Hospitalized	38	65.8	OE	12 (31.6)	2 (5.2)
Zhang et al. [13]	Apr 11	CS	Chia	Hospitalized^*∗*^	102	57.6	OE	2 (2.0)	1 (1.0)
Kumar et al. [14]	May 25	P	India	Hospitalized	45	31.3	NA	NA	1 (2.2)
Karimi et al. [15]	May 18	CS	Iran	Hospitalized	43	56.6	OE	2 (4.7)	3 (7.0)
Xie et al. [16]	Apr 26	P	China	Hospitalized	33	57.6	OE	0 (0)	2 (6.1)
Oncul et al. [17]	Aug 21	CS	Turkey	Hospitalized	359	58.5	Oph	16 (4.5)	NA
Atum et al. [18]	Jul 3	P	Turkey	Hospitalized	40	41.4	OE	10 (25.0)	3 (7.5)
Li et al. [19]	Sep 17	P	Hong Kong	Hospitalized	49	57.1	OE	0 (0)	4 (8.2)
Xia et al. [20]	Mar 12	P	China	Hospitalized	30	54.5	OE	1 (3.3)	1 (3.3)
Tostamann et al. [21]	Apr 25	P	Netherland	Healthcare workers	90	NA	Q	31 (34.4)	NA
Abrishami et al. [22]	Jun 22	CS	Iran	Hospitalized	142	62.6	SL	92 (64.8)	NA
Bostanci Ceran et al. [23]	Jun 6	CS	Turkey	Hospitalized	93	39.4	OE	20 (21.5)	NA
Chen et al. [24]	May 18	P	China	Hospitalized	535	44.0^†^	Tel; Q	27 (5)^§^	NA
Liang and Wu [25]	Mar 18	P	China	Hospitalized	37	NA	OE	3 (8.1)	1 (2.7)
Guan et al. [26]	Feb 28	R	China	Hospitalized and outpatient	1099	47.0^†^	Charts	9 (0.9)	NA
Savastano et al. [27]	Dec 23	P	Italy	Hospitalized	50	69.6	OE	5 (10)	4 (8)
Alizadehsani et al. [28]	Nov 28	P	Iran	Patients	123	52.0	OE	2 (1.6)	NA
Dutescu et al. [29]	Nov 25	P	Germany	Hospitalized	18	66.3	OE	5 (27.8)	5 (27.8)
Boz et al. [30]	Nov 23	CS	Turkey	Hospitalized	50	58.3	SL	9 (18.0)^§^	NA
Meduri et al. [31]	Nov 19	P	Italy	Hospitalized	29	77.1	Q; OE	10 (34.5)	0 (0)
Kaya et al. [32]	Sep 30	P	Turkey	Patients	32	52.8	OE	0 (0)	5 (16.0)
Hanege et al. [33]	Oct 23	CS	Turkey	Patients	38	48.8	OE	0 (0)	21 (55.3)
Pirraglia et al. [34]	Oct 15	CS	Italy	Hospitalized	43	70.0^†^	OE	3 (7.0)^§^	0 (0)
Cavalleri et al. [35]	Aug 17	CS	Italy	Hospitalized	172	64.2	Q; OE	40 (23.3)	NA
Schettino et al. [36]	Sep 30	P	Italy	Hospitalized	190	64.6	Q	17 (8.9)	NA
Mahmoud et al. [37]	Sep 15	P	Egypt	Hospitalized	28	51.8	OE; SL	10 (35.7)	8 (28.6)
Shemer et al. [38]	Sep 14	P	Israel	Hospitalized	16	58.7	OE	3 (18.8)	0 (0)
Lee et al. [39]	Sep 7	R	Korea	Hospitalized	103	47.4	Tel; photo	22 (21.4)	NA
Arora et al. [40]	Aug 31	CS	India	Hospitalized	75	55.7	OE	0 (0)	18 (24.0)
Rokohl et al. [41]	Aug 21	P	Germany	Hospitalized	108	37.9	Q	75 (69.4)	NA
Sindhuja et al. [42]	Jul 24	R	India	Hospitalized	127	38.8^†^	OE	12 (9.4)	NA

Num: number; pts: patients; s/s: symptom/sign; P: prospective case series; CS: cross-sectional; R: retrospective case series; NA: nonapplicable; OE: ocular examination; Q: questionnaire; Oph: ophthalmoscopy; SL: slit lamp; Tel: telephone; ^*∗*^including healthcare workers; ^†^median age; ^§^including conjunctivitis only.

**Table 3:** Clinical features of ocular manifestations among COVID-19 patients.

First author	*N*	Classification of ocular s/s (number of pts)	Pts with ocular s/s as the first s/s (*n*, %)	Prop of pts with ocular s/s among those with severe pneumonia	Prop of pts with ocular s/s among those with mild-mod pneumonia
Seah	17	Redness (1); chemosis (1)	0 (0%)	NR	NR
Hong	56	Follicular conjunctivitis (15); redness (15); ocular pain (15); dryness (15); discharge (15); itching (15); foreign body sensation (15)	6 (10.7%)	9/32 (28.1%)	6/24 (25%)
Zhou	121	Redness (3); itching (5); discharge (2); foreign body sensation (2); watering (3)	NR	7/58 (12.1%)	1/63 (1.6%)
Guemes-Villahoz	301	Follicular conjunctivitis (35); redness (35); discharge (33); foreign body sensation (12); watering (15)	NR	10/65 (15.4%)	25/236 (10.6%)
Wu	38	Follicular conjunctivitis (12); redness (12); discharge (12); chemosis (12); watering (12)	1 (2.6%)	8/15 (53.3%)	4/23 (17.4%)
Zhang	102	Follicular conjunctivitis (2); redness (1); chemosis (1); watering (1)	NR	NR	NR
Karimi	43	Follicular conjunctivitis (1); foreign body sensation (1)	1 (2.3%)	NR	NR
ONcul	359	Conjunctivitis (10); redness (9); discharge (6); watering (5); photophobia (4); subconjunctival hemorrhage (5); chemosis (2)	NR	4/65 (6.2%)	12/294 (4.1%)
Atum	40	Follicular conjunctivitis (10)	NR	NR	NR
Xia	30	Follicular conjunctivitis (1)	NR	0/9 (0%)	1/21 (4.8%)
Tostmann	90	Ocular pain (31)	NR	NR	NR
Abrishami	142	Redness (23); itching (12); watering (33); foreign body sensation (4); periorbital pain (5); irritation (19)	0 (0%)	14/28 (50%)^*∗*^	30/114 (26.3%)^*∗*^
Bostanci Ceran	93	Follicular conjunctivitis (8); redness (20); itching (13); watering (9); chemosis (3); discharge (6); photophobia (15); itching (13); burning sensation (7); foreign body sensation (5)	NR	NR	NR
Chen	535	Redness (27); watering (55); itching (53); discharge (52); dryness (112); foreign body sensation (63); photophobia (16); blurred vision (68)	4 (0.7%)^*∗*^	NR	NR
Liang	37	Follicular conjunctivitis (3); redness (3)	NR	NR	NR
Guan	1099	Redness (9)	NR	NR	NR
Savastano	50	Follicular conjunctivitis (5)	NR	2/16 (12.5%)	2/34 (5.8%)
Alizadehsani	123	Follicular conjunctivitis (2)	NR	NR	NR
Dutescu	18	Redness (5); chemosis (5)	NR	5/5 (100%)	0/13 (0%)
Boz	50	Follicular conjunctivitis (9); watering (9); itching (8); photophobia (4); burning sensation (5); blurred vision (2)	0 (0%)	NR	NR
Meduri	29	Foreign body sensation (3); ocular pain (3); dryness (2); watering (5); hyperemia (7); chemosis (1); discharge (2)	0 (0%)	NR	NR
Pirraglia	43	Follicular conjunctivitis (3); redness (3); hypertensive retinopathy (4); AMD (2); DR (1); chorioretinitis (1)	NR	0/15 (0%)	3/28 (10.7%)
Cavalleri	172	Follicular conjunctivitis (40); rendess (26); watering (23); foreign body sensation (17); itching (12); discharge (4); eyelid swelling (5)	24 (14.0%)	NR	NR
Schettino	190	Nonspecific ocular symptoms (17)	NR	NR	NR
Mahmoud	28	Follicular conjunctivitis (10); redness (10)	NR	7/15 (46.7%)	1/13 (7.7%)
Shemer	16	Active conjunctival injection (3); irritation (5); foreign body sensation (5); discharge (1)	5 (31.3%)^*∗*^	NR	NR
Lee	103	Redness (7); irritation (5); ocular pain (3); blurred vision (6); watering (2); itching (4)	0 (0%)	NR	NR
Rokohl	108	Burning sensation (39); watering (37); redness (28); itching (20); eyelid swelling (15)	NR	NR	NR
Sindhuja	127	Follicular conjunctivitis (8); redness (8); burning sensation (1); watering (1); eyelid swelling (1)	3 (2.4%)	NR	12/127 (9.4%)

*N:* total number of COVID-19 patients; pts: patients; s/s: symptom/sign; Prop: proportion; mod: moderate; NR: not reported; *∗*including conjunctival congestion only.

## Data Availability

The data used to support the findings of this study are available from the corresponding author upon request.

## Abbreviations

COVID-19: Coronavirus disease 2019
SARS-CoV-2: Severe acute respiratory syndrome coronavirus 2
ACE2: Angiotensin-converting enzyme 2
s/s: Symptoms/signs
RT-PCR: Reverse transcriptase-polymerase chain reaction
PRISMA: Preferred Items for Systematic Reviews and Meta-Analyses
OR: Odds ratio
CI: Confidence interval.
